# Voice in the algorithmic era: how perceived algorithmic control influences gig workers’ voice behavior

**DOI:** 10.3389/fpsyg.2025.1637658

**Published:** 2026-01-12

**Authors:** Ting Liang, Yufei Zhang, Dan Xiang, Lingtong Zhu

**Affiliations:** 1 School of Accounting, Southwestern University of Finance and Economics, Chengdu, China; 2 School of International Business, Southwestern University of Finance and Economics, Chengdu, China

**Keywords:** gig workers, perceived algorithmic control, perception of algorithmic fairness, voice behavior, voice endorsement

## Abstract

Existing studies have focused on gig workers’ voice behavior and the mechanisms through which it is expressed via official and unofficial channels, the underlying logic of voice behavior generation, particularly its operational pathways under algorithmic control remains under-investigated. Drawing on fairness heuristic theory, this paper proposes a moderated mediation model to explore the effect of perceived algorithmic control on gig workers’ voice behavior and its boundary condition. Based on the questionnaire data of 260 gig workers, the empirical results indicate that perceived algorithmic control significantly and positively predicts gig workers’ voice behavior by enhancing their perception of algorithmic fairness. Furthermore, voice endorsement moderates the relationship between perception of algorithmic fairness and voice behavior. Complementing these findings, in-depth interviews with 22 gig workers provide a more nuanced understanding of how individuals construct fairness perceptions under algorithmic control and how these perceptions inform their decisions to engage in voice behavior.

## 1 Introduction

With the rapid development of the platform economy, work arrangements on gig platforms have increasingly blurred the boundaries between freelance and traditional employment (Duggan et al., 2020), relying heavily on the outsourcing of classic managerial functions to algorithmic systems (Kellogg et al., 2020). Within this context, algorithms have gradually evolved into the infrastructural governance mechanism through which platforms manage gig workers, reshaping traditional labor relations and situating workers in environments characterized by intense digital surveillance and constant change (Möhlmann et al., 2021). Scholars have conceptualized gig workers’ comprehensive perceptions of how platform algorithms exert real-time dynamic control over the online labor process through standardized guidance, tracking evaluation and behavioral constraint as perceived algorithmic control (Pei et al., 2021), which has been shown to be a key antecedent of their behavioral outcomes (Wiener et al., 2021; Zhu et al., 2024).

Existing research has documented multiple behavioral consequences of perceived algorithmic control. On the positive side, it can enhance service performance (Pei et al., 2021), work engagement (Lin et al., 2025) and continued intentions (Cram et al., 2022; Wiener et al., 2021). On the negative side, high algorithmic control may undermine job autonomy (Liu et al., 2021), proactive behaviors (Pei et al., 2021) and innovative actions (Liu et al., 2021). Some studies further reveal a double-edged sword effect, suggesting that perceived algorithmic control may both heighten and diminish work engagement (Zhang et al., 2024), influence turnover intention (Chen et al., 2024), and exert complex effects on service and safety performance (Zhu et al., 2024; Chen et al., 2025). While these studies have advanced the literature, the question of how perceived algorithmic control shapes gig workers’ voice behavior remains insufficiently examined.

Voice behavior, defined as constructive, improvement-oriented expression that goes beyond formal role expectations (Van Dyne et al., 2003), serves as a critical channel for platform workers to offer input and engage in process improvement, and is especially salient within the emerging labor structure of gig work. Existing studies on voice in gig platforms primarily focus on three aspects: (1) platforms’ use of algorithmic and proceduralized feedback interfaces to restrict meaningful dialog, thereby suppressing institutionalized voice channels (Gegenhuber et al., 2021; Kougiannou and Mendonça, 2021); (2) gig workers’ active use of social media groups and instant messaging tools to build peer networks and communities that foster vibrant informal voice spaces for information sharing, mutual support, and coordinated collective action (Maffie, 2020; Walker, 2021; Zhou and Pun, 2024; McDaid et al., 2023); and (3) workers’ strategic interventions in algorithm-driven organizational processes through speaking up, challenging, or supplementing algorithmic decisions, and, in some cases, initiating collective actions such as coordinated log-offs to counter algorithmic management (Cameron and Rahman, 2022; Wood and Lehdonvirta, 2021; Karanović et al., 2021). While these studies highlight the complexity and agentic nature of gig worker voice, systematic explanations of the individual-level psychological mechanisms remain limited, and theoretical exploration of how platform management features, particularly perceived algorithmic control, shape voice behavior is still unexplored.

To address this gap, drawing on Fairness Heuristic Theory (Lind, 2001), this study proposes perception of algorithmic fairness as a mediating mechanism. The procedural, distributive, and interactional fairness cues conveyed through perceived algorithmic control may help gig workers form positive overall fairness judgments, thereby strengthening their willingness to speak up. Furthermore, platform feedback on voice, namely the level of voice endorsement, serves as a critical signal of whether workers perceive their input as valued (Burris, 2012; Jia et al., 2025). A high level of voice endorsement not only reinforces individuals’ sense of trust and self-esteem, further reinforcing the positive effect of perception of algorithmic fairness on voice behavior.

**Figure 1:**
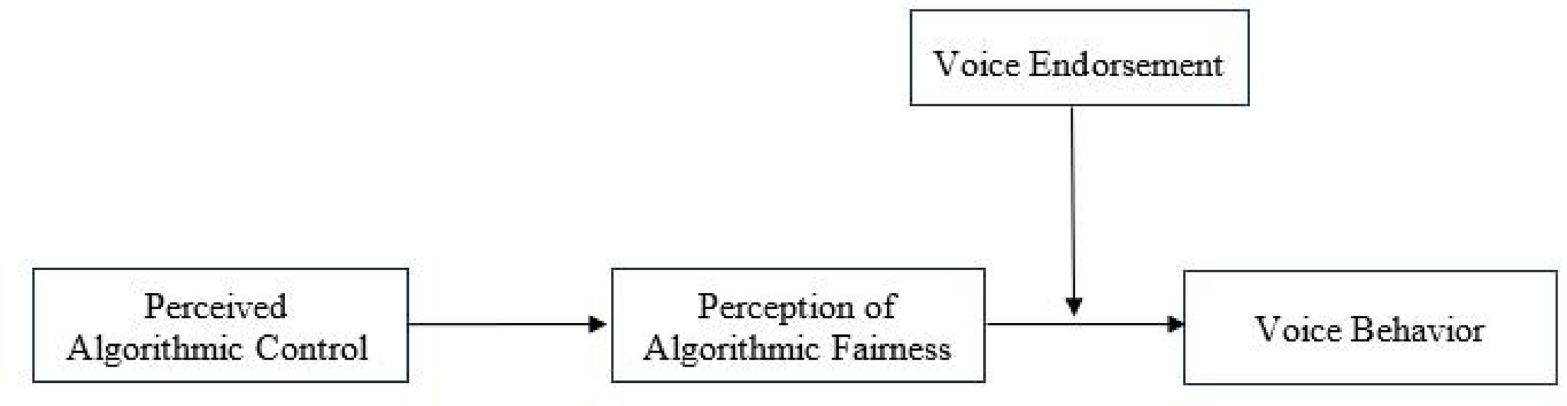
Theoretical model.

In summary, drawing on fairness heuristic theory, this study proposes a moderated mediation model (see Figure 1). This study contributes to the literature in three main ways. First, by investigating the pathway through which perceived algorithmic control affects gig workers’ voice behavior, it extends the research boundary of perceived algorithmic control in the domain of work behavior. Second, by identifying the mediating role of perception of algorithmic fairness, it illuminates the psychological mechanism through which perceived algorithmic control shapes worker behavior. Third, by examining the moderating role of voice endorsement, this paper enriches the boundary conditions that shape the process by which perceived algorithmic control affects voice behavior of gig workers.

## 2 Theory and hypotheses

### 2.1 Perceived algorithmic control and voice behavior

Algorithmic control refers to a form of digital management practice in the gig economy, whereby online labor platforms, based on embedded technical rules and standardized procedures, utilize AI algorithms driven by big data to autonomously execute control functions over gig workers who are dispersed across time and space (Graham et al., 2017; Wood et al., 2019; Pignot, 2023). Building on this foundation, Pei et al. (2021) defined perceived algorithmic control as gig workers’ comprehensive perception of how platform algorithms exert real-time, dynamic control over the online labor service process through standardized guidance, tracking evaluation and behavioral constraint.

In algorithm-driven gig work, interactions between workers and platforms heavily rely on systematic and automated rules. Research indicates that perceived algorithmic control is not merely a supervisory mechanism but can also function as a “technical support partner” by offering clear path planning and real-time feedback, thereby assisting workers in understanding and achieving performance goals (Pei et al., 2021; Chen et al., 2025). Against this backdrop, we anticipate that perceived algorithmic control can promote gig workers’ voice behavior. Existing empirical studies provide preliminary evidence for a positive relationship between the two. First, prior research has shown that perceived algorithmic control can enhance gig workers’ work engagement (Chen et al., 2024; Lin et al., 2025). Work engagement, characterized by vigor, focus, and dedication, activates individual resources and strengthens the motivation to improve work contexts, thereby facilitating voice behavior (Parker et al., 2010; Bindl et al., 2012; Liu et al., 2021). Second, perceived algorithmic control can also increase gig workers’ job embeddedness (Liang et al., 2025), which reflects the alignment, connectedness, and perceived sacrifices between individuals and their work context. Highly embedded employees are more concerned with the long-term development of the organization and are more willing to offer constructive suggestions (Kiazad et al., 2015; Tan et al., 2019; Tabrizi et al., 2023). Based on this, the following hypothesis is proposed:

*Hypothesis 1*: Perceived algorithmic control is positively related to voice behavior.

### 2.2 The mediating role of perception of algorithmic fairness

Perception of algorithmic fairness refers to an individual’s subjective evaluation of whether an algorithmic system adheres to principles of fairness in data processing, decision-making, and outcome delivery (Wei et al., 2021). In the context of the gig economy, gig workers often operate in environments characterized by information asymmetry and cognitive constraints, making it difficult to conduct a comprehensive and rational assessment of algorithmic fairness (Jones-Jang and Park, 2023). According to FHT, when information is limited or contexts are uncertain, individuals are more likely to rely on salient and accessible cues to make heuristic fairness judgments (Lind, 2001; van den Bos et al., 1997). The theory emphasizes that experiences of procedural, distributive, and interactional fairness constitute the core basis for an individual’s overall fairness judgment (Long, 2004).

In algorithmic management contexts, perceived algorithmic control provides gig workers with these three critical fairness cues. First, algorithmic standardized guidance delivers procedural fairness cues by providing clear, consistent, and transparent work standards (Pei et al., 2021; Chen et al., 2025), enabling workers to better understand the basis of algorithmic decisions. Second, algorithmic tracking evaluation conveys interactional fairness cues through real-time, specific, and credible performance feedback (Wiener et al., 2021), reflecting the system’s respect and attention toward workers. Third, algorithmic behavioral constraint provides distributive fairness cues via clearly defined and consistently enforced incentive and penalty mechanisms (Griesbach et al., 2019), allowing workers to perceive a balance between effort and reward. Since these three types of cues originate from the same authoritative system (platform algorithms) and are presented continuously and synchronously in daily work, gig workers can readily integrate them into an overall perception of algorithmic fairness.

Fairness Heuristic Theory further suggested that once individuals form a positive overall fairness judgment, they enter a “group mode,” characterized by greater acceptance of authority and rules, enhanced identification, self-esteem, and trust, and an increased tendency to engage in pro-social behaviors that benefit the organization (Lind, 2001; Long, 2004). In algorithmic management contexts, perceiving algorithms as fair leads workers to regard platform algorithms as reliable and worthy of cooperation, thereby strengthening their motivation to contribute suggestions for collective benefit and encouraging constructive voice behavior (Walumbwa et al., 2012; Duan et al., 2007). Empirical studies also indicate that fairness perception, as an individual’s perceived organizational fairness, is a key organizational factor stimulating voice behavior (Bashshur and Oc, 2015; Colquitt et al., 2001). Therefore, this study posits that perceived algorithmic control, by providing gig workers with procedural, interactional, and distributive fairness cues, fosters their overall perception of algorithmic fairness, which in turn enhances prosocial motivation and promotes voice behavior. Based on this, the following hypothesis is proposed:

*Hypothesis 2*: Perception of algorithmic fairness mediates the relationship between perceived algorithmic control and voice behavior.

### 2.3 The moderating role of voice endorsement

Voice endorsement embodies the platform’s positive feedback and value affirmation toward individuals’ voice behavior (Ni et al., 2024), representing the organization’s acceptance and recognition of the suggestions raised by individuals (Liang et al., 2024). Specifically, this construct encompasses three primary forms: psychological acknowledgment of the suggestion’s value, practical implementation of the suggestion into work routines, or escalation of the suggestion to higher-level authorities to promote its adoption (Burris, 2012; Jia et al., 2025).

First, a high level of voice endorsement signals that platforms effectively accept and implement the suggestions put forward by individuals (Lam et al., 2019; Jia et al., 2025), which enhances gig workers’ trust in the platform’s decision-making processes. For gig workers with perception of algorithmic fairness, they can understand the standards and process of voice adoption, and trust makes them more actively participate in voice behavior, thus more willing to share their ideas. Second, for gig workers with perception of algorithmic fairness, a high level of voice endorsement makes them feel that their ability and value are recognized by the organization (Zhang et al., 2020; Wang et al., 2024). This recognition elevates individuals’ self-esteem and self-efficacy to a certain extent, motivating them to respond positively to challenges and be more inclined to put forward further constructive suggestions. Moreover, organizational acknowledgment enhances individuals’ confidence and stimulates a proactive pursuit of personal growth and development. When gig workers perceive the idiosyncratic competitiveness of colleagues, they often experience a potential status threat, prompting greater efforts to participate in voice behavior to enhance their capabilities and ultimately maintain or improve their standing and competitiveness within the organization (Liu N.-T. et al., 2022; Liu P. et al., 2022). Based on this, the following hypothesis is proposed:

*Hypothesis 3*: Voice endorsement moderates the relationship between perception of algorithmic fairness and voice behavior.

### 2.4 The moderated mediation model

As discussed above, the preceding discussion on the mediating role of perception of algorithmic fairness aims to reveal the psychological mechanisms through which perceived algorithmic control influences gig workers’ voice behavior. The discussion of the moderating role of voice endorsement further clarifies the conditions under which perception of algorithmic fairness exerts a stronger impact on voice behavior. On the basis of the above analysis, this study further proposes a moderated mediation model. Specifically, voice endorsement indicates that individual’s suggestions are positively evaluated and adopted by the platform. This process enables gig workers to perceive the value and effectiveness of their voice, thereby enhancing their self-efficacy and sense of accomplishment. At the same time, it demonstrates the practical significance of their suggestions to the platform, which fosters their self-esteem and strengthens their trust in the platform. When perceived algorithmic control activates gig workers’ perception of algorithmic fairness and thereby promotes voice behavior, high levels of voice endorsement further boost their self-esteem and trust, making them more willing to offer constructive suggestions. Therefore, when the platform voice endorsement level is high, the positive effect of perceived algorithmic control on gig workers’ voice behavior through perception of algorithmic fairness is further strengthened. Based on this, the following hypothesis is proposed:

*Hypothesis 4*: Voice endorsement moderates the indirect effect of perceived algorithmic control on gig workers’ voice behavior via perception of algorithmic fairness, such that the indirect effect is stronger when voice endorsement is high.

## 3 Research design

This study employs a sequential explanatory mixed-methods design (Creswell and Plano Clark, 2018; Zhang et al., 2019) to investigate the relationship between perceived algorithmic control and gig workers’ voice behavior. Phase 1 (Quantitative Study, *N* = 260) empirically tested our hypothesized model, including the mediating role of perception of algorithmic fairness and the moderating role of voice endorsement. This phase establishes the generalizability and statistical significance of the hypothesized relationships and provides the primary evidence for the moderating effect of voice endorsement. Building directly on the quantitative results, Phase 2 (Qualitative Study, *N* = 22) conducted in-depth interviews to serve an explanatory function with respect to the main pathway from perceived algorithmic control to voice behavior via perception of algorithmic fairness. The convergence of quantitative and qualitative evidence on this pathway provides triangulation, while the emergence of “job insecurity” as an additional psychological mechanism illustrates complementarity by extending the initial model centered on perception of algorithmic fairness.

## 4 Quantitative study

### 4.1 Participants and procedure

The target population of this study consisted of gig workers in China, specifically food-delivery riders and ride-hailing drivers (e.g., Didi drivers). A mixed-method approach combining online and offline survey distribution was adopted. To ensure data validity, participants were required to be currently working as a food-delivery rider or a ride-hailing driver. No minimum tenure threshold was imposed during recruitment. Respondents were asked to report their work tenure on the focal platform.

Online questionnaires were distributed via the Credamo data collection platform. Within Credamo, we targeted respondents who were currently working as food-delivery riders or ride-hailing drivers, and we used platform settings to restrict multiple submissions from the same account and IP address. The survey link was released in several waves to reduce the likelihood that the same users would repeatedly receive the invitation. Offline questionnaires were distributed in person by trained research assistants at locations where gig workers commonly gather, such as dedicated rest stations for food-delivery riders and charging stations for drivers. Research assistants briefly introduced the purpose of the study, confirmed that potential participants were currently working as food-delivery riders or ride-hailing drivers, and then invited them to complete the questionnaire on site on a voluntary basis. No recruitment posters or other passive methods were used, all offline participants were recruited through direct, face-to-face invitations.

All respondents participated anonymously and received a small material reward upon completion. A total of 306 questionnaires were collected (241 online and 65 offline). To ensure data quality and participant attention, strict quality control was applied to exclude invalid questionnaires, such as those with unrealistically short completion times or straight-lining response patterns. Ultimately, 260 valid responses (84.97% of the initial sample) were included in our empirical analysis. To assess potential sampling bias between online and offline recruitment channels, we conducted a series of homogeneity tests. Independent-samples t-tests revealed no significant differences in age (*t* = −0.668, *p* = 0.504) or work tenure (*t* = −0.381, *p* = 0.704) between the two subsamples. However, chi-square tests indicated significant differences in gender [*χ*^2^(1) = 4.47, *p* = 0.034], marital status [*χ*^2^(1) = 4.48, *p* = 0.034], and education level [*χ*^2^(4) = 42.86, *p* < 0.001], but not in job type [*χ*^2^(1) = 0.59, *p* = 0.441]. To address these demographic differences and mitigate potential confounding effects, we included all these variables as control variables in our subsequent regression analyses to account for these pre-existing differences, thereby ensuring the robustness of our findings.

### 4.2 Measures

All measures used have been validated in previous research. Given that all administered items were in Chinese, translation and back-translation procedures were followed to ensure the quality of translations (Brislin, 1986). Specifically, the items were first translated into Chinese by a bilingual researcher and then translated back into English by another independent bilingual scholar. The two versions were compared, and minor discrepancies were resolved through discussion to ensure the Chinese items accurately reflected the original meanings. Each measure used a 5-point Likert-type scale ranging from “strongly disagree” to “strongly agree.”

#### 4.2.1 Perceived algorithmic control

Perceived algorithmic control was measured using Pei et al.’s (2019) scale. The 11-item scale has three dimensions: (1) Standardized guidance (four items, example item: “Algorithms intelligently assigns my workload”); (2) Tracking evaluation (four items, example item: “Algorithms track my geographic position in real time”); (3) Behavioral constraint (three items, example item: “Algorithms grade and rank me within the platform based on my job performance”). Cronbach’s *α* was 0.907.

#### 4.2.2 Voice

Three items from Lebel (2016) were used to measure voice behavior in gig workers. An example item was, “Speak up with ideas for new work-related policies and procedures.” Cronbach’s α was 0.747.

#### 4.2.3 Perception of algorithmic fairness

To assess perception of algorithmic fairness, we used Colquitt’s (2001) measure. The 20-item scale has four dimensions: (1) Distributive justice (four items, example item: “The platform’s outcome reflect the effort I have put into my work”); (2) Procedural justice (seven items, example item: “I have been able to express my views and feelings regarding the algorithmic procedures of the gig platform”); (3) Information justice (five items, example item: “The gig platform has been candid in communication with me”); (4) Interpersonal justice (four items, example item: “The gig platform treated me in a polite manner”). Cronbach’s α was 0.949.

#### 4.2.4 Voice endorsement

We adapted two items from Ni et al.’s (2024) voice endorsement measure. An example item was, “I have the feeling that the platform will find my advice valuable.” Cronbach’s *α* was 0.812.

#### 4.2.5 Control variables

Following established practices in organizational research (Becker, 2005; Chen et al., 2025), we included six demographic and occupational variables as controls to mitigate potential confounding effects: gig workers’ gender, age, marital status, education level, job type, and work tenure.

## 5 Results

### 5.1 Descriptive statistics and correlations

Table 1 depict the descriptive statistics and coefficients among study variables for the samples. See Table 1 for details, perceived algorithmic control was significantly correlated with voice behavior (*r* = 0.462, *p* < 0.01) and perception of algorithmic fairness (*r* = 0.709, *p* < 0.01). Perception of algorithmic fairness was significantly correlated with voice behavior (*r* = 0.639, *p* < 0.01).

**Table 1:** Means, standard deviations, correlations, and reliabilities of studied variables.

Variables	Mean	SD	1	2	3	4	5	6	7	8	9	10
1. Gender	1.270	0.442	—									
2. Age	3.070	1.273	−0.106	—								
3. Marital status	1.580	0.494	−0.054	0.467**	—							
4. Education level	3.120	1.021	0.175**	−0.086	0.080	—						
5. Job type	1.690	0.462	−0.166**	−0.005	0.177**	−0.067	—					
6. Work tenure	3.174	2.743	−0.021	0.284**	0.283**	0.083	0.130*	—				
7. Perceived algorithmic control	4.059	0.605	0.038	0.033	0.163**	0.156*	0.061	0.059	(0.907)			
8. Voice behavior	3.603	0.782	−0.01	0.021	0.117	0.024	0.095	0.106	0.462**	(0.747)		
9. Perception of algorithmic fairness	3.794	0.660	0.055	−0.004	0.174**	0.110	0.023	0.035	0.709**	0.639**	(0.949)	
10. Voice endorsement	3.512	0.937	0.095	0.037	0.069	0.077	−0.059	0.056	0.364**	0.598**	0.716**	(0.812)

*N* = 260. ^*^p < 0.05, ^**^p < 0.01. Cronbach’s alphas are shown in parentheses along the diagonal. SD, standard deviation.

Gender: 1 = male, 2 = female; Marriage: 1 = single, 2 = married; Education level: 1 = middle school or below, 2 = high school, 3 = technical school, 4 = undergraduate degree, 5 = master’s degree or above; Job Type: 1 = part-time, 2 = full-time, Age and working tenure are in years.

### 5.2 Confirmatory factor analysis

Before hypothesis testing, we conducted confirmatory factor analysis using Amos to test the measurement model in both samples. As shown in Table 2, the hypothesized four-factor model (perceived algorithmic control, perception of algorithmic fairness, voice endorsement, and voice behavior) fit the data well (*χ^2^/df* = 1.665, *RMSEA* = 0.051, *SRMR* = 0.057, *CFI* = 0.932, *TLI* = 0.926) and was a significantly better fit than all other alternative models, indicating the four variables were distinct from each other.

**Table 2:** Confirmatory factor analysis.

Models	χ^2^	df	*χ*^2^/df	RMSEA	SRMR	CFI	TLI
Four-factor model:							
The hypothesized four-factor model	955.509	574	1.665	0.051	0.057	0.932	0.926
Three-factor model:							
Combining PAC and voice behavior	1349.473	588	2.295	0.071	0.079	0.865	0.856
Combining voice behavior and voice endorsement	1186.072	588	2.017	0.063	0.064	0.894	0.887
Two-factor model:							
Combining PAC, PAF and voice behavior	1600.168	590	2.712	0.081	0.084	0.821	0.809
One-factor model:							
Combining all variables	1886.728	594	3.176	0.091	0.079	0.771	0.757

*N* = 260. PAC, perceived algorithmic control; PAJ, perception of algorithmic fairness. χ^2^, chi-square; df, degrees of freedom; RMSEA, root mean square error of approximation; SRMR, standardized root mean square residual; CFI, comparative fit index; TLI, Tucker–Lewis index.

### 5.3 Hypothesis testing

Hypothesis development was conducted using ordinary least squares regression and bootstrapping analysis in SPSS 26.0, the results are presented in Table 3. Model 2 revealed that hypothesis 1, which predicted that perceived algorithmic control was positively related to gig workers’ voice behavior, was found to be supported (*β* = 0.593, *t* = 8.076, *p* < 0.001). As Table 3 shows, perceived algorithmic control was positively associated with perception of algorithmic fairness (*β* = 0.762, *t* = 15.465, *p* < 0.001), which in turn was positively related to voice behavior (*β* = 0.765, *t* = 13.18, *p* < 0.001). The results showed that perception of algorithmic fairness mediated the linkage from perceived algorithmic control to voice behavior (*indirect effect* = 0.574, *SE* = 0.096, *95% CI* = [0.390, 0.763]). Thus, hypothesis 2 was supported.

**Table 3:** Ordinary least squares regression.

Variables	Voice behavior				Perception of algorithmic fairness
	Model 1	Model 2	Model 3	Model 4	Model 5
Intercept	3.147^***^(0.326)	1.137^**^(0.383)	0.651^*^(0.315)	0.331(0.321)	0.679^**^(0.257)
Controls					
Gender	0.002(0.113)	−0.020(0.101)	−0.046(0.087)	−0.056(0.083)	0.035(0.068)
Age	−0.030(0.045)	−0.023(0.040)	0.006(0.035)	−0.001(0.033)	−0.038(0.027)
Marital status	0.165(0.116)	0.051(0.104)	−0.055(0.091)	−0.033(0.087)	−0.141^*^(0.070)
Education level	0.007(0.049)	−0.042(0.044)	−0.031(0.038)	−0.013(0.036)	−0.013(0.030)
Job type	0.112(0.110)	0.077(0.098)	0.115(0.085)	0.128(0.080)	−0.050(0.066)
Work tenure	0.023(0.019)	0.022(0.017)	0.024(0.015)	0.018(0.014)	−0.002(0.011)
Independent variable					
Perceived algorithmic control		0.593^***^(0.073)			0.762^***^(0.049)
Mediator					
Perception of algorithmic fairness			0.765^***^(0.058)	0.596^***^(0.084)	
Moderator					
Voice endorsement				0.234^***^(0.055)	
Two-way interaction					
Perception of algorithmic fairness * Voice endorsement				0.183^**^(0.056)	
*R* ^2^	0.026	0.226	0.424	0.17	0.512
*Adjusted R* ^2^	0.003	0.205	0.408	0.13	0.498
*F*	1.136	10.537^***^	26.455^***^	4.92^***^	37.743^***^

Indirect effect	Effect	SE	LLCI	ULCI
Perceived algorithmic control → voice behavior (via Perception of algorithmic fairness)	0.574	0.096	0.390	0.763

*N* = 260. ^*^p < 0.05, ^**^p < 0.01, ^***^p < 0.001. Statistics reported are unstandardized regression coefficients (and standard errors); LLCI, lower level confidence interval tested at 95% significance level; ULCI, Upper level confidence interval tested at 95% significance level.

**Figure 2:**
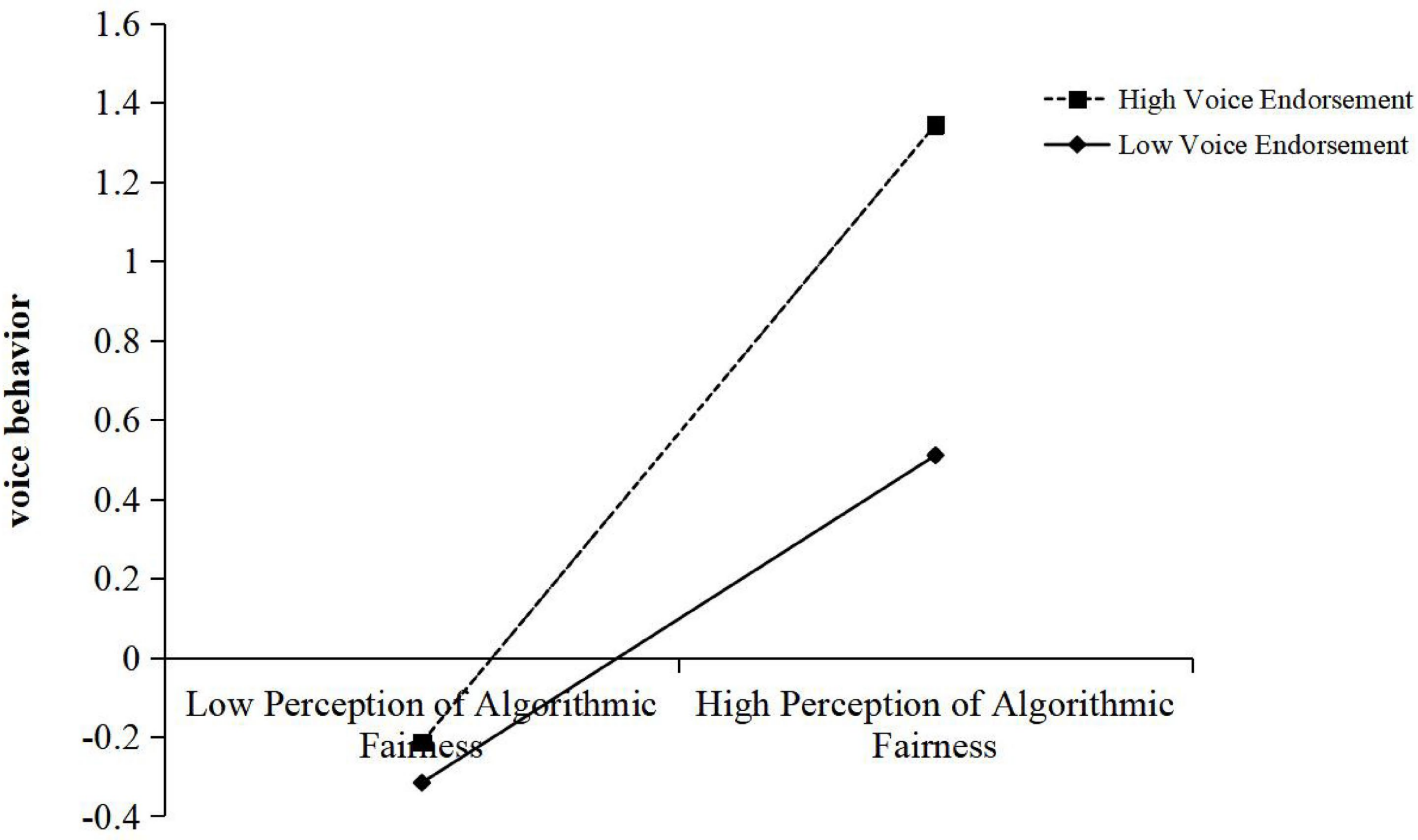
Simple slope of voice endorsement.

Hypothesis 3 predicted that voice endorsement would positively moderate the relationship between perception of algorithmic fairness and voice behavior. The results of model 4 in Table 3 showed that the latent interaction between perception of algorithmic fairness and voice endorsement was significantly related to voice behavior (*β* = 0.183, *t* = 3.282, *p* < 0.01). The simple slope test in Figure 2 shown that the effect of perception of algorithmic fairness on voice behavior was significant when voice endorsement was high (M + 1SD) (*simple slope* = 0.672, *SE* = 0.159, *p* < 0.001), whereas the effect was non-significant at the low level (M-1SD) (*simple slope* = 0.324, *SE* = 0.173, *p* = 0.061). These results support Hypothesis 3.

Following the procedures recommended by Preacher et al. (2007), we utilized the PROCESS macro in SPSS to examine the full moderated mediation model. This analysis was conducted using 5,000 bootstrap resamples to generate 95% bias-corrected confidence intervals. As reported in Table 4, the results revealed that when voice endorsement was high (M + 1SD) (*indirect effect* = 0.504, *SE* = 0.123, *95% CI* = [0.263, 0.737]) and low (M−1SD) (*indirect effect* = 0.255, *SE* = 0.128, *95% CI* = [0.005, 0.512]), the indirect effects of perceived algorithmic control on voice behavior via perception of algorithmic fairness was significantly positive. While, the difference in direct effect between “high” and “low” values of voice endorsement was significant (*indirect effect* = 0.249, *SE* = 0.127, *95% CI* = [0.007, 0.501]). Thus, Hypothesis 4 was supported.

**Table 4:** Moderated mediation results of bootstrapping testing.

Condition	Effect value	SE	Boot 95%CI	
			Lower level CI	Upper level CI
High voice endorsement (M + 1SD)	0.504	0.123	0.263	0.737
Low voice endorsement (M−1SD)	0.255	0.128	0.005	0.512
Difference between high and low	0.249	0.127	0.007	0.501

CI, confidence interval tested at 95% significance level. SE, standard errors.

## 6 Qualitative study

### 6.1 Participants and procedure

This study employed semi-structured in-depth interviews with 22 gig workers. We adopted a purposive sampling strategy to recruit participants, aiming to intentionally recruit information-rich cases that were closely aligned with the research topic. Specifically, we visited locations where gig workers gather, such as food-delivery rider rest stations and ride-hailing driver charging rest areas, approached and invited individuals to participate in the interviews. We focused on two types of workers, specifically food-delivery riders and ride-hailing drivers, because they represent the most typical forms of gig work in China’s platform economy and effectively illuminate the common experiences of platform workers under algorithmic management (Huang, 2023).

Sample characteristics are as follows: Among the 22 interviewees, 21 were male and 1 was female. Age distribution: 20–30 years (*n* = 8), 30–40 years (*n* = 3), 40–50 years (*n* = 10), 50–60 years (*n* = 1). In terms of occupation, 14 were food-delivery riders (Ele.me: *n* = 7; Meituan: *n* = 7) and 8 were ride-hailing drivers (Didi: *n* = 8). Each interview lasted approximately 15–30 min and was transcribed. Table 5 presents detailed background information on the interviewees.

**Table 5:** Background information about the interviewees (*N* = 22).

Participants	Gender	Age group	Occupation	Platform
1	Male	20–30	Food Delivery Rider	Ele.me
2	Male	20–30	Food Delivery Rider	Ele.me
3	Male	20–30	Food Delivery Rider	Meituan
4	Male	20–30	Food Delivery Rider	Meituan
5	Female	40–50	Food Delivery Rider	Meituan
6	Male	20–30	Food Delivery Rider	Meituan
7	Male	20–30	Food Delivery Rider	Meituan
8	Male	40–50	Food Delivery Rider	Meituan
9	Male	20–30	Food Delivery Rider	Meituan
10	Male	40–50	Food Delivery Rider	Ele.me
11	Male	30–40	Food Delivery Rider	Ele.me
12	Male	40–50	Food Delivery Rider	Ele.me
13	Male	40–50	Food Delivery Rider	Ele.me
14	Male	20–30	Food Delivery Rider	Ele.me
15	Male	30–40	Ride-Hailing Driver	Didi
16	Male	50–60	Ride-Hailing Driver	Didi
17	Male	30–40	Ride-Hailing Driver	Didi
18	Male	40–50	Ride-Hailing Driver	Didi
19	Male	40–50	Ride-Hailing Driver	Didi
20	Male	40–50	Ride-Hailing Driver	Didi
21	Male	40–50	Ride-Hailing Driver	Didi
22	Male	40–50	Ride-Hailing Driver	Didi

Prior to each interview, we informed all participants about the study purpose and confidentiality measures and obtained their informed consent. All interviewees were assigned pseudonyms, and interview data were used solely for academic purposes.

Based on the research questions, this study designed an interview outline focusing on three core dimensions: perceived algorithmic control (e.g., “How do you perceive platform management while working on this platform?”), perception of algorithmic fairness (e.g., “Do you think you are treated fairly while working on the platform?”), and voice behavior (e.g., “When you encounter problems at work, do you provide feedback to the platform? Could you give an example of a suggestion you have made?”). Extensive open-ended questions were incorporated to encourage respondents to fully express their lived experiences, capture underlying information and emergent themes, and allow for deeper exploration through follow-up questions, ensuring data richness and depth.

This study employed systematic grounded theory (Strauss, 1987), adopting an inductive coding approach to allow themes to emerge from the data. The specific process involved open coding, axial coding, and selective coding, to gradually construct a theoretical framework. Coding was performed independently by two coders who received training and engaged in discussions to establish unified coding standards. When disagreements arose, coders discussed and reached consensus through deliberation. Representative examples of the three-level coding process, including the correspondence from original interview quotes to each coding level, are displayed in Tables 6–9.

**Table 6:** Perceived algorithmic control: theme and empirical evidence.

Second-order themes	First-order concepts	Sample quotations
Standardized guidance	Order dispatch rationality	One time the platform assigned me an order that someone else had rejected. I only had 2 min left when I received it, and I could only end up exceeding the time limit (Interviewee 3)
		Recently, the orders have been for long distances and the number of orders has been low (Interviewee 5)
		Generally, there’s no such problem with the distance between two orders; the system assigns orders based on proximity (Interviewee 3)
	Qualification certification and dispatch	I have obtained both certificates. Depending on the city’s management level, having these certificates is not always mandatory. I quite agree with the requirement to have both certificates (Interviewee 17)
		Drivers with dual certificates tend to receive more orders, as platforms prioritize dispatching orders to them (Interviewee 20)
	Location and navigation	The system sometimes dispatches orders in the opposite direction (Interviewee 13)
		The platform sometimes provides the wrong location. The route planning during driving is too rigid; often you can only follow the route given by the system, which wastes a lot of time (Interviewee 18)
		Some merchant locations have significant deviations, and addresses are unclear, wasting a lot of time for riders to locate them (Interviewee 2)
Tracking evaluation	Customer feedback mechanism	After each order is completed, customers evaluate our service, providing a reference for the platform assessments (Interviewee 1)
	Activity monitoring	If you have not driven for a long time and then start again, the platform will assign you fewer orders (Interviewee 18)
Behavioral constraint	Performance evaluation system	To get more orders, it depends on the driver’s seniority, quality driver status, and service rating (Interviewee 18)
		A system glitch led to my service points being deducted because I canceled an order over three kilometers away. Deductions affect my order reception. The full score is 490; you can take reserved orders, etc. After my score dropped, my wait time for orders became longer (Interviewee 17)
	Stringent penalty mechanism	The penalty for being late is very intimidating (Interviewee 5)
		There are many point deduction rules; drivers canceling orders counts as a violation (Interviewee 19)

**Table 7:** Perception of algorithmic fairness: theme and empirical evidence.

Second-order themes	First-order concepts	Sample quotations
Distributive fairness	High commission rate	The platform takes a 30% commission (Interviewee 18)
		Didi’s commission is too high, sometimes reaching 38% (Interviewee 20)
	Lack of welfare protection	No social security insurance was purchased; there is accident insurance (paid for by myself). Never reported the social security issue, felt it would be useless to speak up (Interviewee 12)
		Welfare is a complicated matter. It seems like delivery riders just do not have insurance, and I’ve never heard about any insurance (Interviewee 3)
		The welfare is just for show; its small medical insurance is pretty useless, not as good as buying my own. I did not buy it, and probably 95% of drivers do not buy it either (Interviewee 17)
Procedural fairness	Algorithm adjustability	The platform can manually change orders if they are arranged unreasonably (Interviewee 11)
		If the system’s positioning is wrong, Ele.me and Meituan have been improving their systems recently (Interviewee 9)
	Complaint handling mechanism	If a customer files a malicious complaint, we can seek help from the platform for resolution (Interviewee 7)
		Regarding customer complaints, the platform handles them case by case. We have call recordings. If it’s the rider’s fault, they penalize accordingly; if not, the station manager will investigate before handling it (Interviewee 8)
Informational fairness	Perceived rule transparency	I feel the platform’s rules are fairly transparent, the regulations are quite clear, and I somewhat trust the platform. (Interviewee 8)
		The rules and systems are relatively transparent, and the platform’s management is pretty good (Interviewee 12)
	Lack of platform trust	I have looked into this platform’s management model and do not really trust it anymore; there’s too much information asymmetry (Interviewee 3)
		Regarding the platform’s management, I know a bit about it and do not really trust it because it does not feel very friendly toward riders—there’s just no sense of trust between us and the company (Interviewee 4)
Interpersonal fairness	Ineffective customer support	Customer service just reads from a script. Sometimes when we clearly have not broken any rules, they cannot provide valid evidence of the violation (like showing pictures or something), and even say it involves “internal secrets” (Interviewee 15)
	Poor communication channels	Some merchants are slow in preparing meals (Interviewee 3)
	Ineffective appeal mechanism	I previously returned a lost item to a customer, but was falsely accused of demanding a fee for it. I eventually called the platform, hoping for a quick solution to this kind of issue, but in the end, nothing came of it (Interviewee 16)
		Some passengers make malicious complaints, and basically, our appeals as Didi drivers are useless (Interviewee 20)

**Table 8:** Voice behavior: theme and empirical evidence.

Second-order themes	First-order concepts	Sample quotations
Voice mechanism	Blocked feedback channels	I think giving feedback to these outsourcing companies is useless, we can only follow their rules. At most, we can give feedback to the station manager to see if they can solve it. If the station manager cannot solve it, it gets reported up level by level (Interviewee 4)
	Procedural complexity	I gave feedback, they said they would resolve it for me, and in the end, it was resolved, but the process was very convoluted (Interviewee 3)
Voice effectiveness	Perfunctory feedback	Sometimes Didi company sends out some kind of survey questionnaire. It’s basically useless. Later when they sent them, I basically did not answer (Interviewee 20)
	Superficial resolution	I told the station manager about a merchant’s problem, the merchant improved for a few days, then went back to how they were (Interviewee 5)
	Voice ineffectiveness	I suggested that the platform should charge their fees compliantly and should not put the discount on the drivers to attract passengers, but it was no use (Interviewee 20)
		Feedback is just a facade. Even if I’m in the right, the platform will evade the issue. No matter how reasonable I am, the customer is always right (Interviewee 16)
		I’m already used to the platform’s system. Sometimes the system dispatch is particularly unreasonable, but telling the station manager is useless (Interviewee 5)
		Giving feedback to the platform is done by phone. Sometimes I cannot even be bothered to call anymore; the problems do not get solved (Interviewee 17)
	Unchanged outcomes	Once, I was somewhere, the station manager called me saying an order was about to timeout, transferred the order under my name, and told me to confirm delivery, but it had not been delivered to the customer. The customer complained about me, and then the platform fined me 400. I went to the station manager, who said he would resolve it by the end of the month. By the time I left, it still wasn’t resolved (Interviewee 4)

**Table 9:** Job insecurity: theme and empirical evidence.

Second-order themes	First-order concepts	Sample quotations
Employment entrapment	Reluctant choice	Among the Didi drivers around me, they have not found suitable jobs yet. Didi is relatively flexible, and the money is easier to earn compared to other jobs (Interviewee 20)
		This job is part of my long-term plan, I need to support my children (Interviewee 5)
	Age barriers	I’m older now, and it’s not easy to do other jobs (Interviewee 18)
		I’ve reached that age. I’ll retire in a few years anyway, so I’ll just make do with this job (Interviewee 16)
Institutional powerlessness	Contract comprehension barriers	The signed contract is incomprehensible (Interviewee 19)
	Absence of legal safeguards	I have never raised the issue of commission because it is meaningless. It has not exceeded 30%. Only if it does, will there be dedicated personnel to investigate and punish (Interviewee 17)

**Figure 3:**
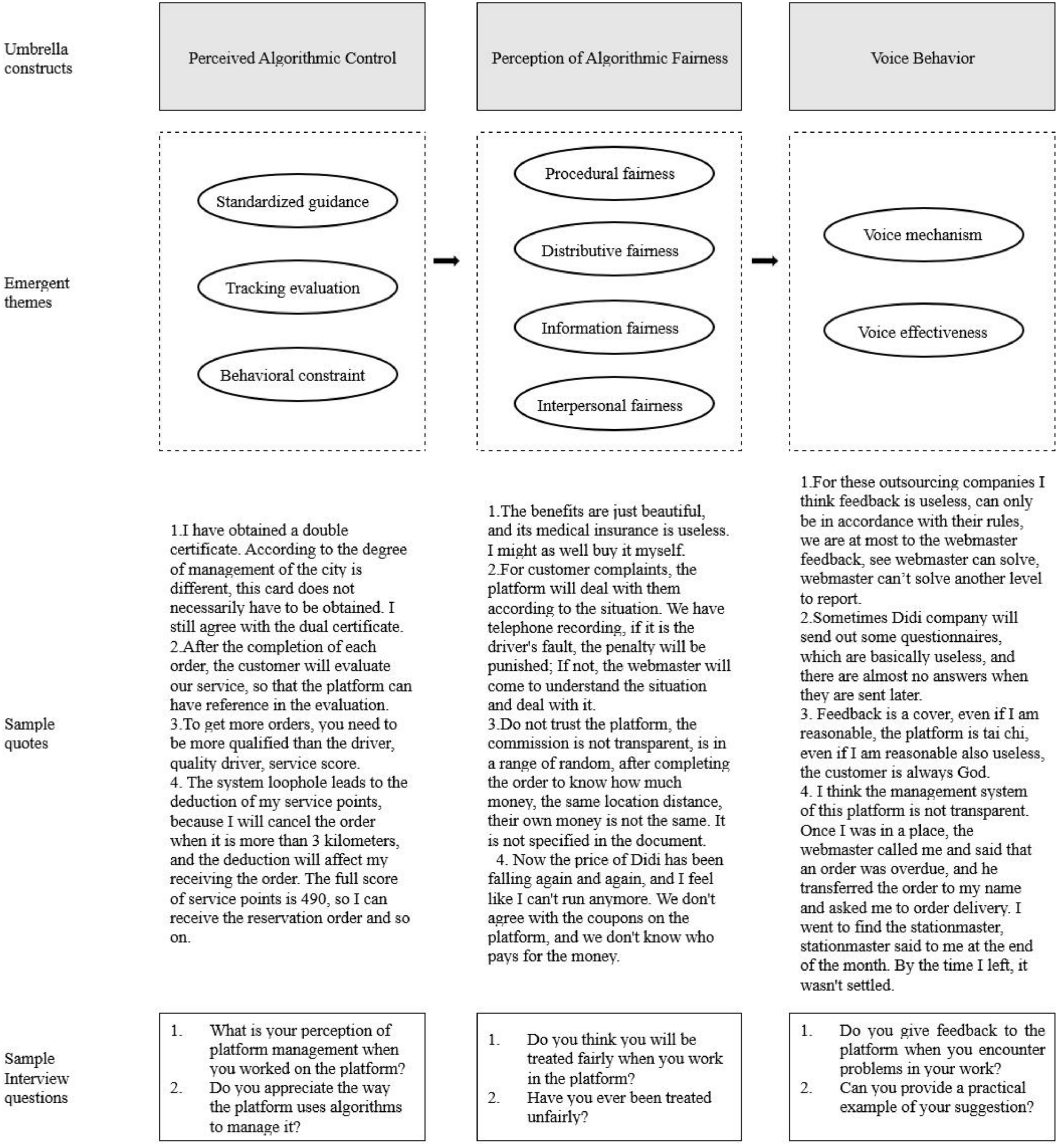
Results of thematic analyses.

Following the practices of Boyatzis (1998) and Owens and Hekman (2012), the analysis ultimately identified nine specific categories under the three core structures of perceived algorithmic control, perception of algorithmic fairness and voice behavior. Figure 3 summarizes the themes that emerged from our data.

### 6.2 Major findings

#### 6.2.1 Gig workers’ subjective perception of algorithmic control significantly shapes their judgment of algorithmic fairness

Perceived algorithmic control, through the dimensions of standardized guidance, tracking evaluation and behavioral constraint, significantly influences gig workers’ perceptions of algorithmic fairness, specifically in the following ways:

1. Standardized guidance and distributive fairness: Algorithmic standardized guidance directly impacts distributive fairness through order assignment quality, order volume distribution, and positioning support. While platforms aim to improve efficiency through order assignment bias and positioning optimization, this practice actually exacerbates resource allocation inequality. For instance, interviewee 20 noted, “Drivers with dual certificates tend to receive more orders, as platforms prioritize dispatching orders to them,” which reduces order opportunities for uncertified workers and reinforces the inequality of distribution rules. Additionally, algorithmic positioning errors (e.g., “Some merchant locations have significant deviations, and addresses are unclear, wasting a lot of time for riders to locate them” [Interviewee 2]) force workers to bear extra costs, further highlighting the imbalance in the platform’s interest distribution mechanism.
2. Tracking evaluation and procedural fairness: The tracking evaluation mechanism, based on customer reviews and work frequency data, ostensibly ensures procedural fairness but, due to the lack of algorithmic transparency and correction mechanisms, actually intensifies procedural unfairness. For example, “After each order is completed, customers evaluate our service, providing a reference for platform assessments.” [Interviewee 1] However, the absence of impartial verification for malicious complaints leads to workers passively accepting unreasonable punishments (e.g., “The penalty for being late is very intimidating” [Interviewee 5]). While Interviewee 7 mentioned, “if a customer files a malicious complaint, we can seek help from the platform for resolution.” The actual appeal process is complex and inefficient, rendering procedural fairness a mere formality.
3. Behavioral constraints and interpersonal fairness: Algorithmic behavioral constraints, through rating systems and service scores, regulate worker behavior, but their stringent punishment mechanisms (e.g., “There are many point deduction rules; drivers canceling orders counts as a violation” [Interviewee 19]) undermine the equality in the platform-worker relationship. Platforms regard workers as algorithmic control subjects rather than partners. Interviewee 17 stated, “Didi’s prices keep dropping, making it hard to continue. We drivers do not agree with the platform’s discount coupons. We never receive subsidies for them and do not know who bears the cost.” This one-sided constraint weakens the respect and collaboration in interpersonal fairness, leading workers to perceive unfairness.
4. The interaction between standardized guidance and tracking evaluation further undermines information fairness. For example, the ambiguity of order assignment rules and commission ratios (e.g., “I do not trust the platform; commission rates aren’t transparent. They’re randomly determined within a range, and we only know our earnings after completing an order” [Interviewee 17]) leads to information asymmetry, making it difficult for workers to anticipate their income and intensifying distrust in the platform (e.g., “I have looked into this platform’s management model and do not really trust it anymore; there’s too much information asymmetry” [Interviewee 3]).

#### 6.2.2 Gig workers’ subjective perceptions of algorithmic fairness play a critical role in driving their voice behavior

Perceptions of algorithmic fairness influence both the mechanisms and outcomes of voice through four dimensions: distributive, procedural, information, and interpersonal fairness.

1. Distributive fairness and voice mechanisms: High commission rates (e.g., “Didi’s commission is too high, sometimes reaching 38%” [Interviewee 20]) and nominal welfare programs (e.g., “The welfare is just for show; its small medical insurance is pretty useless, not as good as buying my own. I did not buy it, and probably 95% of drivers do not buy it either” [Interviewee 17]) triggered by distributive unfairness prompt workers to express their concerns and advocate for their rights. However, entrenched power structures within the platforms (e.g., “I think giving feedback to these outsourcing companies is useless, we can only follow their rules. At most, we can give feedback to the station manager to see if they can solve it. If the station manager cannot solve it, it gets reported up level by level” [Interviewee 4]) blocks voice channels, leading most workers to adopt passive adaptation (e.g., “I’m already used to the platform’s system. Sometimes the system dispatch is particularly unreasonable, but telling the station manager is useless” [Interviewee 5]).
2. Procedural fairness and voice effectiveness: The absence of procedural fairness (e.g., inefficient appeal mechanisms) directly weakens the effectiveness of voice behavior. Interviewee 15 noted, “Feedback is just a facade. Even if I’m in the right, the platform will evade the issue. No matter how reasonable I am, the customer is always right.” Even when workers propose reasonable suggestions (e.g., “I suggested that the platform should charge their fees compliantly and should not put the discount on the drivers to attract passengers, but it was no use “[Interviewee 20]), the platform lacks motivation for improvement, creating a vicious cycle.
3. Information fairness and voice effectiveness: The lack of transparent information feedback mechanisms prevents workers from tracking the progress of their voice behavior. For example, interviewee 5 mentioned, “I told the station manager about a merchant’s problem, the merchant improved for a few days, then went back to how they were.” This recurrence of unresolved problems directly undermines the effectiveness of voice efforts.
4. Interpersonal fairness and voice mechanisms: Platforms treat workers as “algorithmic control subjects” rather than partners. Interviewee 17 stated, “Didi’s prices keep dropping, making it hard to continue. We drivers do not agree with the platform’s discount coupons. We never receive subsidies for them and do not know who bears the cost.” This unequal relationship makes voice mechanisms lack two-way interaction. When workers propose suggestions, they often encounter responses were ignored, rendering the mechanism ineffective in addressing genuine concerns.

#### 6.2.3 Gig workers’ job insecurity is a key constraint inherent in the nature of their working pattern

This insecurity stems from their high dependence on platforms and the lack of legal protection. Against the backdrop of limited employment options, age growth and skill deficiencies render gig work a primary means of livelihood for many “informal employment” groups. Interviewee 18 noted, “I’m older now, and it’s not easy to do other jobs,” and Interviewee 20 said, “Didi is relatively flexible, and the money is easier to earn compared to other jobs,” highlighting their reliance on platforms for basic survival. However, this structural dependence on platforms significantly exacerbates workers’ economic vulnerability, trapping them in a passive situation. Additionally, the absence of legal protection further deepens gig workers’ helplessness. On one hand, complex platform contracts with information asymmetry leave many interviewees “unable to comprehend the contracts they sign” [Interviewee 19], making it difficult to seek legal remedies when their rights are violated. On the other hand, current laws lack clear provisions on critical issues such as platform commissions and task allocation, leaving workers without effective institutional safeguards. For instance, Interviewee 17 pointed out, “I have never raised the issue of commission because it is meaningless. It has not exceeded 30%. Only if it does, will there be dedicated personnel to investigate and punish.” The platform’s behavior lacks effective supervision. Against this backdrop, most workers hold a pessimistic attitude toward the complaint and feedback mechanism, believing that feedback is meaningless, eventually leading to collective silence and passive acceptance of unfair situations.

### 6.3 Discussion

Building upon the quantitative findings, this study further incorporates qualitative analysis to uncover the subtle and complex psychological mechanisms linking gig workers’ subjective perceptions to their voice behavior. Specifically, it elucidates how perceived algorithmic control influences their willingness to voice through perception of algorithmic fairness. The interview results reveal that gig workers commonly perceive the management control of platform algorithms in terms of standardized guidance, tracking evaluation and behavioral constraint, which in turn shape their perceptions of algorithmic fairness. This cognitive process not only enhances their motivation to express opinions but also enhances their initiative to interact with the platform. It is worth noting that the study also finds that the common job insecurity of gig workers may pose potential interference to their voice behavior, which provides important clues for exploring the mechanism of individual psychological factors in the context of algorithmic governance in the future.

## 7 Conclusion

Drawing on fairness heuristic theory, this study systematically investigates the relationship between perceived algorithmic control and gig workers’ voice behavior, further revealing the underlying psychological mechanisms and boundary conditions of this relationship. Based on survey data from 260 gig workers, the empirical results show that perceived algorithmic control significantly enhances gig workers’ voice behavior, with perceptions of algorithmic fairness serving as a mediating mechanism. Moreover, voice endorsement strengthens the positive effect of perception of algorithmic fairness. To deepen our understanding of the processes and contextual factors underlying these relationships, we conducted in-depth interviews with 22 gig workers. The qualitative findings illustrated the pathway from perceived algorithmic control to voice behavior through fairness perception. More importantly, they identified job insecurity as an emergent theme that repeatedly arose in workers’ narratives, pointing to a potentially important contextual factor for future research.

### 7.1 Theoretical contribution

First, this paper extends the research on perceived algorithmic control to the domain of proactive behaviors, and enriches the literature on the relationship between perceived algorithmic control and voice behavior. Existing research on perceived algorithmic control has predominantly focused on its impact on gig workers’ job attitudes and performance outcomes, such as work engagement and turnover intention (Lang et al., 2023; Zhang et al., 2024; Yu et al., 2024). However, whether perceived algorithmic control can stimulate workers’ proactive behaviors aimed at improving organizational processes remains underexplored. At the same time, although recent studies have begun to examine voice behavior among gig workers, they tend to emphasize the channels (Bucher et al., 2024) and forms (Gegenhuber et al., 2021; Karanović et al., 2021) through which voice is expressed, while offering limited insight into why gig workers choose to voice and what intrinsic motivations drive such behaviors. In the context of algorithm-driven digital labor, this study systematically analyzes how perceived algorithmic control influences gig workers’ voice behavior through cognitive processes. In doing so, it not only broadens the theoretical application of perceived algorithmic control in the field of proactive behaviors but also provides a new theoretical perspective and analytical framework for understanding how gig workers demonstrate willingness to participate under platform governance structures.

Second, this paper reveals the psychological cognitive mechanism of perceived algorithmic control on voice behavior. While prior research has established that algorithmic control shapes workers’ attitudes and behaviors (Cram et al., 2022; Zhang et al., 2024), it has largely overlooked the underlying psychological processes. Drawing on fairness heuristic theory, this study introduces perception of algorithmic fairness as a mediating variable, clarifying how individuals form overall fairness perceptions based on previous experiences of procedural, distributive, and interpersonal fairness, thereby activating or inhibiting voice behavior. This paper not only illuminates the cognitive mechanisms underlying this influence process, but also substantiates the explanatory power of fairness heuristic theory in the context of digital labor, thereby enriching its application in emerging organizational forms.

Third, this paper clarifies the boundary conditions of gig workers’ voice behavior under perceived algorithmic control. By introducing voice endorsement as a moderator, this paper demonstrates that when platforms respond positively to worker suggestions, workers perceive their input as valued, which amplifies the positive effect of perception of algorithmic fairness on voice behavior. This finding not only identifies important boundary conditions in the influence path of perceived algorithmic control, but also provides a theoretical basis for understanding how platform organizations promote gig worker participation through voice feedback mechanism. Moreover, it extends the contextual understanding of voice behavior formation mechanism within the field of organizational behavior.

### 7.2 Practical implication

First, platforms should balance efficiency with human-centered care in the design of algorithmic control to enhance gig workers’ acceptance of algorithmic rules. This paper finds that gig workers are not entirely passive recipients of algorithmic management; rather, they cognitively evaluate the fairness embedded in algorithmic systems and decide whether to make suggestions accordingly. Therefore, platforms should avoid overemphasizing control and efficiency when designing task allocation and performance evaluation algorithms. Instead, they should enhance the transparency and explainability of algorithmic rules. For example, clearly explaining the rationale behind platform rules and the mechanisms for individual performance evaluation can foster a stronger sense of fairness among workers, thereby increasing their willingness to participate in voice.

Second, platforms should strengthen gig workers’ positive perceptions of algorithmic fairness by improving organizational mechanisms. The findings indicate that perceived algorithmic control does not necessarily provoke resistance; the key lies in whether workers perceive the algorithm as “fair.” Therefore, platforms should not only optimize the logic of algorithmic systems but also establish corresponding institutional mechanisms to support communication and participation. For instance, incorporating worker representatives in rule-making processes, organizing regular feedback sessions, and publishing algorithm transparency reports can all enhance the credibility and legitimacy of platform governance, thereby promoting stability and constructive interactions of gig labor relations.

Third, platform managers should establish responsive voice mechanisms that include timely acknowledgment, implementation updates, and explanations for non-adoption. This study finds that voice endorsement significantly amplifies the positive effect of perception of algorithmic fairness on voice behavior, indicating that workers’ willingness to continue speaking up depends on whether their voice is “heard.” Therefore, platforms should establish both official and unofficial voice feedback mechanisms, which should not only offer channels for expression, but also provide timely responses regarding whether suggestions are adopted and, if not, explaining the rationale. Such practices help cultivate an inclusive and respectful interaction culture, enhancing workers’ organizational identification and willingness for long-term collaboration.

### 7.3 Limitations and future directions

First, this paper adopts a cross-sectional data collection approach, which limits the ability to capture the dynamic evolution of variables. Particularly in identifying causal relationships among perceived algorithmic control, perception of algorithmic fairness, voice behavior, and voice endorsement. Future research could adopt a longitudinal design using multi-wave data to analyze dynamic processes. This would allow for a clearer understanding of the temporal sequencing and interaction pathways among these variables, thereby enhancing the rigor of causal inference.

Second, regarding sample selection and data quality, the current study focused primarily on two typical platform occupational groups: food-delivery riders and ride-hailing drivers. We did not impose a minimum tenure threshold or specific content-based attention checks during recruitment. We did, however, apply procedural quality controls, such as screening for unrealistically short response times and straight-lining patterns, and included tenure as a control variable in our analyses. Nevertheless, this narrow occupational focus and the absence of stricter inclusion criteria may limit the generalizability and precision of the findings. Future research should expand the scope to include other gig roles, such as online content creation and home services, and consider implementing minimum experience requirements and explicit attention checks to further improve external validity and data reliability.

Third, in the qualitative interviews, the interviewed gig workers generally expressed concerns about job insecurity, which is related to high dependency and lack of labor security. However, the quantitative part of the current study has not yet sufficiently incorporated this psychological state into the theoretical model and tested it empirically. Future research is recommended to further focus on the generation mechanism of insecurity in the context of digital platform governance, as well as its potential impact on gig workers’ trust and voice behavior. Such efforts would provide a more comprehensive describe the psychological adaptation and behavioral reactions of gig workers in an algorithm-dominated environment.

Fourth, the generalizability of the qualitative findings is constrained by the gender imbalance in the interview sample, with only one female participant, reflecting the male-dominated on-site demographics of the sampled industries. While the core theoretical mechanisms under investigation are not predicated on gender, future research should purposively include more female gig workers to explore potential gender-based nuances in their perceptions and experiences.

## Data Availability

The raw data supporting the conclusions of this article will be made available by the authors, without undue reservation.
